# Return to Work after Common Mental Disorders: A Qualitative Study Exploring the Expectations of the Involved Stakeholders

**DOI:** 10.3390/ijerph17186635

**Published:** 2020-09-11

**Authors:** Jessica Scharf, Peter Angerer, Gesine Müting, Adrian Loerbroks

**Affiliations:** 1 Institute of Occupational, Social and Environmental Medicine, Centre for Health and Society, Faculty of Medicine, University of Düsseldorf, Universitätsstr. 1, 40225 Düsseldorf, Germany; jessica.scharf@med.uni-duesseldorf.de (J.S.); peter.angerer@uni-duesseldorf.de (P.A.); gesine.mueting@med.uni-duesseldorf.de (G.M.); 2 Mannheim Institute of Public Health, Social and Preventive Medicine, Mannheim Medical Faculty, Heidelberg University, 69117 Heidelberg, Germany

**Keywords:** common mental disorders, return to work, occupational physician, qualitative study

## Abstract

Common mental disorders (CMDs) are risk factors for long-term sickness absence and unemployment. Therefore, return-to-work (RTW) processes have been introduced to facilitate the return of employees. As the success of RTW processes is considered to be determined by the cooperativeness of the involved stakeholders, we aimed to investigate the views of those stakeholders to disclose potentially diverging expectations. Qualitative interviews were conducted (08/2018-04/2019) among five stakeholder groups: returnees with a diagnosed CMD who were eligible for a RTW process; health care professionals nominated by the returnees; supervisors, colleagues and occupational physicians (the latter three groups were not nominated by the returnees). In total, 24 returnees, 13 health care professionals, 13 occupational physicians, 9 supervisors and 9 colleagues were interviewed (68 interviews in total). Potentially diverging expectations of the stakeholders related to whether diagnoses need to be disclosed by returnees. Agreement existed in terms of the need for a trustful relationship between employees and occupational physicians to initiate a RTW process early. As the understanding of all stakeholders’ viewpoints is one of the main factors promoting a successful RTW, we explored the expectations of those involved in the RTW process. One implication of our findings is to strengthen the role of occupational physicians, who could coordinate the return process.

## 1. Introduction

Common mental disorders (CMDs), such as depression and anxiety disorders, are highly prevalent in industrialized countries and predict reduced work ability, long periods of sickness absence, early retirement due to illness and unemployment [1,2,3]. Therefore, CMDs are associated with high costs for social security systems as well as for the affected individuals [4]. Apart from financial consequences, the psychological well-being of the individuals with CMD may be further reduced by the loss of job-related resources due to sick leave such as one’s responsibilities, recognition from work and social networks. Therefore, effective measures to reintegrate employees with CMD, so-called return-to-work (RTW) processes, are needed. While best practice guidelines for employers to reintegrate employees with a CMD have been presented [5,6], the effectiveness of most RTW interventions has been described as poor [7]. However, the combination of work-related interventions and clinical interventions compared to clinical interventions alone has been found to be associated with a reduced number of days on sick leave [7]. Therefore, the integration of clinical treatment and work-directed measures shows promising results. 

In combining those clinical and work-related intervention approaches, different professions would have to collaborate. Relevant stakeholders comprise, besides the returning employees, therapists (e.g., psychotherapists, psychiatrists), physicians (e.g., general practitioners, occupational physicians) as well as human resources managers (e.g., personnel management, supervisors) and colleagues of the returnees. Additionally, the representative for employees with disabilities or members from private social networks (e.g., family members, friends) can be included if the returnee wishes this support [8]. Previous research has suggested though that the cooperation of these different professions and stakeholders to achieve a successful RTW has been suboptimal so far [9,10,11]. While collaboration among different stakeholders is considered important by members of the different professions themselves, it is rarely effectively implemented in practice and needs to be extended [11]. In particular, the cooperativeness and communication intensity seems to be poor due to lack of time and due to the unawareness of the roles and responsibilities of the involved stakeholders [9]. A study of Stegmann & Schröder (2016) suggests that a successful RTW process is characterized by the ability to adopt the perspective of the involved actors of the RTW process, which means that all parties are empathic. A key aspect of empathy is to compare one’s own and others’ perspectives to reach an agreement on collective aims [12]. Another concept usually considered distinct from empathy is perspective-taking. Perspective-taking refers to the ability to understand how other individuals perceive a situation from their specific perspective on a cognitive level while empathy focusses on emotional processes [13]. Nevertheless, when comparing the concepts of empathy and perspective taking, perspective taking results in greater success (e.g., reaching one’s own goals) than empathy during (competitive) negotiations [13]. As the discussions between the returning employee and the supervisor in the context of return to work can take the form of negotiations, the ability of perspective taking is relevant to clarify underlying interests and to generate creative solutions [14].

Therefore, due to the importance of the awareness and consideration of the mutual concerns, wishes and expectation of different stakeholders related to the RTW process, the present study will explore the views of different stakeholders. Those insights are needed to foster mutual understanding, communication and collaboration among the involved parties and may thereby improve the RTW process. 

## 2. Materials and Methods

### 2.1. Qualitative Study Design

First, we carried out qualitative interviews among patients/returnees in formal employment suffering from CMDs (i.e., affective disorders according to ICD-10 including anxiety disorders, depressive disorders and stress-related disorders). Patients had to be entitled to be offered a RTW process, which is the case after six weeks on sick leave throughout a 12-month period in Germany. A topic guide was used to discuss the shortly impending return to work of the individuals as well as their expectations from involved stakeholders (see below for details). Patients nominated, whenever applicable, their psychiatrist, psychological psychotherapist, general practitioner as well as occupational physician for interviews that were to address the return to work of the patient (after exempt from their pledge of secrecy by the patients). Independently of the interviewed patients, other stakeholders of the RTW process were also recruited and interviewed. Those included employees from different sectors without and with a leadership position (hereafter labeled colleagues and supervisors, respectively). Furthermore, as we aimed to investigate the role of the occupational physician within the RTW process, we also interviewed an additional sample of occupational physicians (i.e., not nominated by patients). 

In line with the participants’ preferences, interviews could be held either via telephone or in personal contact in the clinics/practices, at the workplace or at participants’ homes. There has been no evidence suggesting that information value differs by the mode of interviewing (face-to-face versus telephone interviews) [15]. A compensation of 50€ was offered to every participating psychological psychotherapist and physician. Interviewing of patients, supervisors and colleagues was continued until thematic saturation was achieved, which implies that the inclusion of additional individuals will likely not yield any new topics and findings [16]. The number of recruited psychiatrists, psychological psychotherapists and physicians was contingent upon the number of releases from confidentiality that the patients signed. Interviews with additionally recruited occupational physicians as well as supervisors and colleagues were likewise held until thematic saturation was reached. Our study has been approved by the Ethics Committee of the Medical Faculty of the Heinrich-Heine-University of Düsseldorf (study registration number: 6210R).

### 2.2. Recruitment Process

Patients were recruited in two cooperating clinics: a psychosomatic clinic with inpatients and a psychiatric daycare clinic. These clinics were accessible due to previous cooperation. Patients were selected and approached for interviews based on two inclusion criteria: (1) diagnosis of a CMD (i.e., affective disorders according to ICD-10 including anxiety disorders, depressive disorders and stress-related disorders) and (2) the patient’s intention to return to work of after the rehabilitation. Furthermore, we recruited patients who had recently returned to work after sick leave due to a CMD, who had been offered an official RTW process and still had RTW meetings. This recruitment built on occupational physicians of our institute who are involved in the RTW processes of different companies and institutions.

Psychiatrists, psychological psychotherapists as well as occupational and outpatient physicians, who were exempt from their pledge of secrecy by the patients, were either contacted and interviewed within the clinics or contacted and informed about the study by post and then interviewed by phone. 

### 2.3. Data Collection and Analyses

We developed specific topic guides for each type of study population. The topic guide for patients inquired after their own experiences (e.g., “Which expectations regarding your return to work do you have?”, “Who could assist you during your return?”, “Do you have any ideas or suggestions on how the return process could be optimized?”), wishes and fears pertaining to their RTW. Colleagues and supervisors were asked how they would react to a colleague/employee returning after long-term sick leave after mental illness (e.g., “Do you have any expectations of the returning employee regarding communication and occupational tasks?”, “Do you think that the returning employee has any expectations of you?”, “To what extent do you see the support of your colleague as your task?”). As the interviewed colleagues and supervisors may not have any experiences with colleagues/employees on long-term sick leave (due to mental illness), we developed a case vignette based on the prior qualitative interviews with patients. Thus, the interviewed colleagues and supervisors either could draw on and report their own experiences or respond to the case vignette. The psychiatrists, psychotherapists and general practitioner were asked to reflect on the RTW process of their specific patient (e.g., “Would you have needed any further information in order to be able to better prepare the patient’s return?”) as well as to share their general perspective on the RTW process and on how to improve it (e.g., “From your perspective, do patients often receive letters of invitation for a return to work interview?”, “To what extent did you already have personal contact to companies or company doctors regarding the return to work of patients?”). Additionally, recruited occupational physicians were invited to share their experiences as well as their suggestions for improvement pertaining to the RTW process (e.g., “Which role did you play in the return to work process?”, “Do you think there is any need to speak to other health care professionals regarding the return to work of an employee with CMD?”). Participants also completed a standardized questionnaire to collect sociodemographic and occupational data.

Data collection was carried out between August 2018 and April 2019. The qualitative interviews were either held face-to-face or via telephone and were digitally recorded as well as transcribed. All interviews were conducted and analyzed by Jessica Scharf (JS), who has an educational background in public health and experience in occupational health research, including qualitative research [17]. The material was analyzed using the software package MAXQDA 12. Main categories of the qualitative analyses were derived from the topic guides (i.e., deductive coding). Subcategories were added by inductively coding the interview material. Separate analyses were carried out for each study population (five groups). During data collection as well as between the two rounds of data analysis, the categorization and results were discussed within the study team. The demographic and occupational data were gathered for descriptive purposes and was thus only analyzed in terms of frequencies (i.e., *n* and %) as well as means and standard deviations (SDs).

## 3. Results

### 3.1. Study Population

In summary, we interviewed five stakeholder groups comprising returnees, therapists, occupational physicians, supervisors and colleagues. Qualitative results are therefore presented for those five groups. References of quotes are presented in parenthesis in the results section and listed in full detail in a table attached as online supplement. Abbreviations used for the stakeholder groups are presented as follows: OP: occupational physician, P: patient (P1 is the first patient interviewed), T(P1): therapists (nominated therapist of patient one), S: supervisor and C: colleagues. For the presentation of statistical results the results of the returning employees are presented in Table 1 and the stakeholder groups of therapists and occupational physicians are summed up as health care professionals (Table 2). Additionally, the results for the study sample of supervisors and colleagues are presented together (Table 3) so that the statistical results are summed up for three study samples. In total, we interviewed 68 stakeholders of the RTW process. The first study population consists of returning employees (*n* = 24), the interview duration ranged between 16 min and 59 min with a mean duration of 36 min (SD = 12). In total, 26 health care professionals were interviewed, with a mean interview duration of 29 min (SD = 9) varying between 14 and 50 min. The study population of supervisors and colleagues comprises 18 participants with interviews lasting between 19 and 64 min with a mean of 38 min (SD = 12).

Table 1 shows the characteristics of the study population of the returning employees. In total, 24 patients were interviewed with a mean age of 48.8 (SD = 9.6) years and 10 patients were female. More than half of this study population had a vocational training and almost one-third worked in the secondary sector and the remaining in the tertiary sector. Almost all patients reported to have a permanent contract and most worked 20 to 40 h per week. All of the patients intended to work more than 20 h per week after their return while seven of them would like to work less. Most patients stated that their work predominantly builds on mental activity and about one-fourth reported to work equally mentally and physically. The actual mean work ability of the patients compared to the highest work ability ever reached was reported to be moderate. At the time of the interview, half of the patients were treated in the inpatient rehabilitation clinic, one-third was staying at the daycare rehabilitation clinic and the remainder had already returned to the workplace. Almost all patients reported to suffer from depressive episodes or burnout. More than two-thirds of the interviewees self-reported to have been on sick leave for at least six months and all patients had contact to at least one health care professional concerning their mental illness. The patients reported a medium own mental health and little feelings of shame due to their own mental illness. On average, the patients would rather not disclose their illness to their colleagues (for exact data, see Table 1). 

The study sample of health care professionals comprises psychiatrists, psychotherapists, general practitioner and occupational physicians (Table 2). The health care professionals were 51.6 (SD = 10.4) years old on average and about half of the participants were female. About half of the study population comprises therapists with different professional backgrounds (e.g., psychiatrists, psychotherapists) and the other half reported to be an occupational physician. The mean work experience of the interviewed health care professionals in their current profession is 15.6 (SD = 9.0) years.

As presented in Table 3, the mean age of the 18 interviewed supervisors and colleagues is 50.3 (SD = 10.1) years with half of the population being female and half of the population holding a leadership position. More than half of the employees reported to have a university degree and all of the interviewees are working in the tertiary sector. More than two-thirds are working between 20 and 40 h a week and 13 of the 18 interviewed reported that the working predominantly involves mental activity. None of the employees reported a formal sick leave due to mental illnesses during the past 12 months and good mental health was reported. The mean current work ability was rated to be almost the best ever reached work ability. The colleagues and supervisors did rather not agree that they would avoid personal contact to colleagues with a CMD and partly agreed that support from colleagues for employees with a CMD in the company would be present. In the case that supervisors and colleagues themselves would have a CMD, they reported a moderate willingness of disclosing their illness to other colleagues.

### 3.2. Results from the Qualitative Interviews

**Figure 1:**
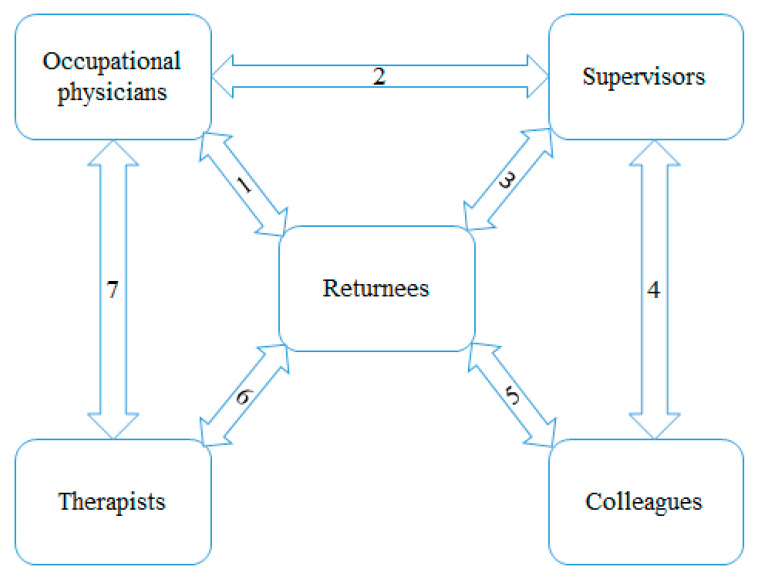
Paths of mutual expectations of different stakeholders involved in the return to work processes.

Figure 1 summarizes the findings pertaining to the expectations of the different interviewed stakeholders involved in the RTW process. The study population of returnees represents the center of the figure as the returnee is the only stakeholder who may be in contact with all other stakeholders. There is no connection between the colleagues of the returnee and the therapists of the returnee. We did not intend to investigate those relations, which are usually not existent. All other shown pathways connecting the different stakeholders are described below in terms of mutual expectations.

Path 1: 

Returnees have the following expectations towards occupational physicians:

Occupational physicians are predominantly seen as officials who have to verify the disease and are not seen as supporters, who could help employees when returning to the workplace. Therefore, returning employees expect the occupational physicians to draw attention to their potential to provide support (1, see Supplementary Materials, Table S1: Quotes.). Employees often know the occupational physicians from pre-employment examinations or through vaccinations. The potential supporting activities during the RTW process are generally not obvious for sick-listed employees. Especially in smaller companies (generally defined by less than 50 employees), the occupational physician is often not present and is therefore not seen as a contact person in the event of illness (2). A previous contact or positive experiences with the occupational physician in the past could contribute to a trustful relationship and a higher chance that the occupational physician is seen as a helpful contact person by the employees. If the occupational physician is involved in the RTW process, the returnees expect her/him to explore which interventions (such as changing the workplace, reducing working time, etc.) are feasible in the company. This should be done early in the RTW process in order to avoid raising unfounded expectations among the employee. 

Returnees further wish that interventions developed through conversation between the returnee and occupational physician should also be assessed by the physician for feasibility against the background of the physician’s knowledge of the company structures (3). In this context, occupational physicians should ensure that supervisors with decision-making authority are involved in the RTW process so that the measures discussed are not rejected by those responsible. The returnees expect their occupational physicians to speak about the individual situation of the returnee with the respective supervisor(s) after having clarified the contents in advance. As returnees reported that they do not really know which measures would be the best for a successful return to their workplaces, they wished that occupational physicians should derive suggestions from previous re-integrations and share this experience with them (4). From the returnees’ perspective, the adherence with agreements is seen as important for the successful reintegration. Therefore, returnees wished occupational physicians to support the enforcement of measures if supervisors do not adhere to the agreements (5). Employees expect occupational physicians to provide follow-up appointments for aftercare.

Occupational physicians have the following expectations towards returnees:

Occupational physicians see their own responsibility, as well as the returnees’ motivation and interest in the return to work as important factors influencing the success of the RTW process (6). As occupational physicians are neither notified when an employee is on long-term sick leave (i.e., six weeks or more) nor are they informed of the employees’ diagnosis, an early contact between occupational physician and employee needs to be initiated by the employees themselves. Occupational physicians believed that this (early) contact to them ensures that measures can be initiated, before the employee’s return (7). According to occupational physicians a prerequisite for success of the RTW process is to pursue open communication. Hidden agendas on the employee’s side (e.g., returnees hoping for early retirement) often complicate communication between occupational physician and employee. Ambiguities about the actual intention of the different stakeholders can endanger a successful RTW process (8). Occupational physicians also argued that a returnee’s request for change should be formulated realistically and certain willingness to compromise must be present (9). The willingness to actually change working conditions as agreed on during RTW meetings must exist among returnees. Furthermore, occupational physicians see the need that returnees should understand their supervisors and colleagues who had to cover extra work by taking over the returnee’s duties during sick leave. They therefore may show limited understanding for the returnee (10).

Path 2:

Supervisors have the following expectations towards occupational physicians:

The main claim of supervisors regarding the occupational physicians relates to their visibility in the company. Specifically, the occupational physicians should be present in the company, offer consultation hours and try to call attention to their range of activities. As a result, occupational physicians should be able to work preventively and establish a good relationship with the employees they look after. Supervisors hope that this will be a point of contact to which the employees turn and speak openly about health- and work-related problems (11).

Occupational physicians have the following expectations towards supervisors: 

The interviewed occupational physicians expect an appreciative attitude towards the returnees and an honest interest in the resumption of the employee in the sense of the supervisors’ duty to care for their subordinates (12). To fulfill this duty, occupational physicians expect supervisors to either have knowledge or at least to have the willingness to acquire knowledge about mental illnesses. Additionally, occupation physicians want supervisors to deal with the disease characteristics and especially to recognize mental disorders as an illness (long-term illness, possible restrictions due to medication, etc.) (13). Based on this understanding of the illness, the communication between supervisors and returnees should take place without prejudice. Solutions should be constructive and, if necessary, supervisors should support the implementation of measures to restructure the working conditions of the returnee (14). Especially, the fact that returnees with a CMD often need more time for their tasks after their return should be accepted (e.g., due to exhaustion, lack of self-confidence, etc.) or even the adaptation of a new range of tasks should be considered (15). Occupational physicians also expect supervisors to strictly comply with agreements pertaining to the initiated measures (16). According to occupational physicians, an empathetic relationship with their employees is necessary to show this considerate behavior (17).

Path 3: 

Returnees have the following expectations towards supervisors:

The returnees expect from their supervisors to take time for conversation and to show honest interest in their personal situation with an open communication style. The first conversation should take place before the return to work (at the latest on the first working day) so that the returnees feel well-received and agreements on work performance can be clarified in advance (18). According to returnees, this first conversation can also help supervisors to find out how resilient the returning person currently is. In this context, returnees expect that supervisors should not put pressure on them and immediately expect the returnees to be as resilient as before their sick leave (19). Therefore, most interviewed returnees wished that supervisors offered a stepwise reintegration opportunity to return to work which allows a slow and controlled enlargement of workload. When employees return with a stepwise reintegration, smaller tasks that can be accomplished during the reduced working hours help to increase self-confidence and productivity (20). If the performance profile of the returnees has changed, supervisors are expected to accept and to adapt the working situation to the new performance profile as well as to work out appropriate measures. Furthermore, according to returnees, supervisors are supposed to accept, if the returnee only wants to communicate about her/his new functioning profile and do not want to disclose the specific illness. As the interviewed returnees often stated that mental disorders are different from physical disorders (in terms of perceptibility of health restrictions), they often did not know how the company (i.e., supervisors) could help with the coping of the disease. Therefore, returning employees often do not know which changes at the workplace can be demanded and are effective, so that returnees would wish for suggestions for measures, which have been useful for previous returnees. These presented measures can then be accepted or rejected by the employee (21). When measures are being discussed, it is important that the superiors clearly communicate to the returnees if measures cannot be implemented. In this way, returnees do not hold expectations that cannot be addressed at all, which would lead to further frustration (22).

Supervisors have the following expectations towards returnees:

Supervisors expect returnees to take on responsibility on their own in terms of informing the company/the supervisors as soon as they know the date of return. From the supervisors’ point of view, this is the only way to prepare for the return and, if necessary, to make adjustments to the workplace and work tasks in advance. Therefore, supervisors see the need for an open communication especially when returnees are suffering from mental illnesses. In order to be able to show understanding for the returnee and to gain understanding from colleagues, it is often expected that returnees communicate the symptoms of the disease. At least problems that affect every day work should be explained as precisely as possible in order to gain understanding by supervisors (23). Furthermore, supervisors stated that they are only able to initiate measures addressing the health restrictions if they know what the specific restrictions are. Even if the diagnosis does not necessarily have to be disclosed, however, the disclosure of symptoms and restrictions often implies that conclusions can be drawn about the specific illness, which is usually not desired by the returnees (24). Apart from work, supervisors expect returnees to seek psychological support. Only if the employees strive to improve their health status, supervisors think that they can show understanding for the returnee. In this context, supervisors themselves do not want to take over the role of the psychotherapist (25). The interviewed supervisors also often stated that they feel overwhelmed by the expectation of the returnees, and that they perceived to be expected to suggest what can be changed at the workplace. As the supervisors do not have a complete understanding of the symptoms and consequences of CMDs, they do not know which measures could be helpful. Therefore, most supervisors see the responsibility to develop specific measures not with themselves, but with the returnees and their therapists. 

Path 4:

Colleagues have the following expectations towards supervisors:

Since the absence of a sick colleague usually implies an additional workload for the remaining colleagues, the colleagues expect their supervisors to inform them as early as possible about how long they should carry out the extra work. From the colleagues’ perspective, the additional burden needs to be limited in time, in order to ensure that the colleagues do not fall ill themselves. In case that a timely restriction cannot be granted by the supervisor, colleagues perceive that it is the supervisor’s duty to communicate to the higher-level supervisors that the scope of tasks of the affected team has to be reduced or also taken over by the supervisor himself/herself (26). Another important point raised by the interviewed colleagues pertained to the recognition from the supervisors for the extra work they are doing to compensate the missing colleague (27).

Supervisors have the following expectations towards colleagues:

Supervisors expect from the returnees’ colleagues to take over the extra work. In addition, the supervisors expect the colleagues to show understanding for the returnee and to accept the possible restrictions of the returnee. Supervisors recognize that this might be difficult for the present colleagues and therefore focus on an open communication in the team (28).

Path 5:

Colleagues have the following expectations towards returnees:

Many of the interviewed colleagues stated that knowledge of the specific disease makes it easier to show understanding for the returnee. In addition, it was often mentioned that specific help can only be offered if the illness and the associated restrictions are disclosed (29). Nevertheless, the colleagues stated that they could only show understanding and offer support over a limited time and would therefore expect a returnee to be able to take over her/his duties again after a certain period of time. This period cannot be precisely defined and depends on when the colleagues feel overworked by the tasks they have to take over from the returnee (30). As long as the colleagues perceive that the returnees actually make an effort to fulfill their working tasks, it is accepted by the other colleagues if the returnee does not complete all the tasks and the colleagues still have to provide support (31).

Returnees have the following expectations towards colleagues:

The interviewed returnees basically expected a positive and friendly return to the team. The returnees would be happy, if their colleagues would welcome them but they were scared about a constant questioning about the nature of their illness (32). Some of the returnees reported to hope for support and a kind of grace period. Others just wanted to return and to continue working (33). In general, the returnees expected their colleagues to accept their health restrictions and that (if the diagnosis was disclosed) a mental illness was perceived as a serious clinical syndrome. Envy or even bullying would impede the returning process (34).

Path 6:

Returnees have the following expectations towards therapists:

The returnees generally expected patience with the therapy progress and the possibility of long-term care (35). In relation to the RTW process, the returnees expected, for example, a good preparation in terms of discussing the disclosure of the illness, preparing how to speak with supervisors and colleagues as well as own coping strategies at the workplace. In addition, (rehabilitation) therapists can also provide support by writing an official letter if, e.g., a job rotation is necessary (36).

Therapists have the following perspectives and expectations of returnees:

The interviewed therapists expect their patients to be willing to take the step back to the workplace and to take responsibility for their return process (37). The success of the therapy is dependent on the honesty and transparency about the own goals of the returnees and their intention to return to the workplace (38). Therapists also expect that patients see that they could also have difficult personal traits, which should be observed by oneself and not only be seen in others. A willingness to change is described as necessary for a successful resumption of work (39). The essential aim of therapy would be the application of measures and behaviors developed in therapy at the workplace by the returnees (40).

Path 7:

Therapists have the following expectations towards occupational physicians:

The interviewed therapists stated that it would be desirable if occupational physicians are present in the company as the contact person for the employees. Regardless of illnesses, the contact can lead to a trustful relation in case of a need for advice. If rehabilitation is initiated by the occupational physician (by the company) the therapists would like to receive work-related information from the occupational physician. This facilitates therapeutic work with the patient due to a more objective evaluation of the working conditions (41). In principle, the participating therapists considered the involvement of the occupational physician to be useful in order to gain more objective insights into the company and to incorporate the company perspective into the preparations for return. However, the subjective point of view of the patient is often sufficient, since the patient’s perception is crucial for the therapy (42). Nevertheless, a trustful and protected support for the returnee by the occupational physician, maintaining confidentiality and acting in the interests of the returning employee, are of particular relevance for the success of the RTW process according to the interviewed therapists. During therapy, therapists often experience that patients have great concerns that the occupational physician represents the employer’s interests and is sharing confidential information. Therapists themselves are unsure whether the disclosure of information about the employee in a conversation with the occupational physician could legally endanger the patient’s employment relationship (43). Furthermore, therapists indicated that even if they would like to talk to an occupational physician, the occupational physicians would usually not speak to psychotherapists or, above all, to social workers, but only to other physicians (psychiatrists). Nevertheless, therapists in rehabilitation clinics lack the time for phone calls or a more detailed discussion with occupational physicians (44).

Occupational physicians have the following expectations towards therapists: 

Since occupational physicians are not informed about sick-listed employees, it would be helpful if the treating therapist informed the patient about the role of occupational physicians in companies and their expertise in RTW processes and, if necessary, suggested that an appointment is made. Occupational physicians would also expect that the therapists would integrate the returnee’s working conditions and arrangements in the therapy and provide information about the RTW process (45). In addition, the (dis)advantages of a participation in the RTW process could be discussed. As it is challenging to completely reorganize the working conditions of an employee, the therapists are expected not to stir up false expectations, such as, that due to the size of the company, alternative workplaces must be available (46). In this context, the returnee’s functional profile should be formulated in as much detail as possible so that potential restrictions are psychologically or medically coherent. Ultimately, if there are too many restrictions, there is always the risk that there is no corresponding job for the returning employee (47).

## 4. Discussion

The present study explored the expectations among different stakeholders involved in the RTW process. A key aspect, mentioned by all stakeholder groups, pertained to the need for open communication about expectations and the aims pursued by the different types of stakeholders during the RTW process. At the same time, all stakeholders acknowledged that an open communication about CMDs is difficult due to the risk of stigmatization. The returnees’ fear and their communication of their performance profile (e.g., work-related limitations) would indirectly disclose their diagnosis. Therefore, a stronger emphasis should be put on the communication between the stakeholders as well as a trustful communication about the changed performance profile of the returnee.

### 4.1. Findings in Light of Prior Research and Implications

The communication between the stakeholders involved in the RTW process has been examined in previous studies. In agreement with our study, it has been found that the different stakeholders have unfulfilled communication needs, which may be not known to the other stakeholders [9,10]. The importance of open communication during the RTW process has therefore also been highlighted by a previous study [12]. In Germany, the RTW process is regulated by law and demands the employer to take measures in order to overcome the returnees incapacity to work as well as to maintain her/his workplace in case that sick leave took longer than six weeks within one year (§ 167 Abs. 2 SGB IX). The employers are also requested to involve other professions and stakeholders in the RTW process, such as the integration offices, workers’ councils and occupational physicians. One study from Germany found that—in addition to unmet communication needs—the cooperation between the occupational physician as well as physicians and therapists of rehabilitation clinics is poor [18]. This is due to the limited presence of occupational physicians in companies leading to a poor bond of trust between employees and occupational physicians. Furthermore, due to the limited presence in the companies, occupational physicians are difficult accessible for therapists. In our study, these aspects were also reported by the psychotherapists and by the supervisors, suggesting that the occupational physicians should establish relationships to the employees already before sickness absence. Otherwise, the low trust of employees in occupational physicians will present a barrier of the RTW process [9]. 

There is agreement regarding the need for an early communication also from the occupational physicians’ and returning employees’ view. Nevertheless, for reasons of data protection, the occupational physicians are not informed about sick-listed employees. Occupational physicians could therefore be proactive by communicating their unique position (i.e., medical confidentiality) to the workforce and to establish a bond of trust. This may lead to an earlier contact to the occupational physician during a sickness absence period, but maybe also enables the occupational physician to detect illness in an early stage, that is, prior to sickness absence [9,19]. Thus, it is suggested, that educational opportunities related to CMDs should be integrated in the training of occupational physicians as well as the development of guidelines pertaining to the prevention of CMDs at the workplace [20]. In Germany, the training regulations for becoming an occupational physician comprise the assessment of mental stress and training on the RTW of employees with CMDs. Nevertheless, the specific teaching content is not yet standardized leading to a broad variance of offered trainings. It is possible that the progressive digitalization and thus digital teaching approaches will increase the comparability of offered trainings and therefore may improve teaching standards. Additionally, since returnees with a CMD on long-term sick leave usually have contact to psychiatrists or psychologists, these health care professionals could highlight the support, which occupational physicians could provide, and advise the returnees to make an appointment with the occupational physician.

The supervisors’ perception of the RTW process has been analyzed by a qualitative study of Lemieux et al. (2011) [21]. In line with our study, the interviewed supervisors mentioned a lack of information regarding the diagnosis of the returnee as hindering factor of the RTW process. As wished by the returnees of our study, Lemieux et al. found that the supervisors also mentioned that meeting the returnees on his/her first day of the return would facilitate the RTW process. During this meeting, expectations of both stakeholders could be discussed and the returnee could be informed of any changes that had taken place within the organization [21]. The lack of knowledge of CMDs was reported to be a hindering factor by the supervisors [21]. Another study reported that the interviewed supervisors had close contact to the workforce and stayed in contact with the employee during sick leave. Furthermore, the intention of the supervisor to take measures to facilitate the employees’ RTW process was positively associated with the duration of the absence period and with the success of the RTW of the employee [22]. 

A meta-synthesis of qualitative research on RTW among employees with CMD found that, amongst others, social support at work and the acknowledgment of the supervisors that work-related factors have contributed to the illness as well as stepwise RTW and reduced working hours are seen as most relevant for returning employees [23]. It is therefore recommended to train supervisors to develop supportive strategies, skills pertaining to communication and listening as well as improving the working conditions [22,24]. The returnees mainly requested from supervisors to provide the opportunity of a stepwise return to the workplace. This stepwise return has already been described as a facilitating factor of RTW processes and is therefore recommended [25]. Most supervisors of our study and from other studies [22,26] agreed that the returnees deserved stepwise introduced tasks and more time for tasks. Nevertheless, this consideration for the returnee is restricted in terms of the time period, as the employee has to fulfill his/her tasks of the working contract according to supervisors interviewed in the present study. Especially, the interviewed potential colleagues of an employee with a CMD emphasized the timely restriction of the additional assumption of tasks. One study among nurses explored views of colleagues with a CMD. The study concluded that the nurses were willing to be supportive, in case they were informed about the sickness and had knowledge about mental disorders [26]. This finding is comparable to our results, as all interviewed colleagues expressed willingness to help a returning colleague, given that they know how to provide support to the returnee and they themselves get not overburdened. Aside from educating the potential colleagues on how to support colleagues with CMDs [26], the interviewed colleagues of our study asked supervisors to distribute the tasks equally and to take over tasks by themselves. Nevertheless, a basic prerequisite for showing understanding and offering support for retuning employees from both the colleagues’ and supervisors’ view pertains to disclosing the work-related limitations related to the disease. 

As returning employees often fear stigmatization, they may not want to disclose their illness and expect that the nature of their illness is known once they share information on disease-related limitations [27,28]. Furthermore, employees may refuse to disclose their illness, if their CMD is partly attributable to their relationship with their supervisor. As the support from supervisors can be positively associated with a successful RTW of the employees (see above), missing support and unsolved conflicts could complicate RTW. Previous research showed that the decision process of disclosing a CMD is complex and the outcome of the decision is influenced by many factors, most of which cannot be influenced by the returnee [29]. As these factors are dependent on the specific circumstances of the employee, recommendations pertaining to the (extent of) disclosure can only be made on an individual basis. This consideration could be a taken over by occupational physicians within a RTW process. Additionally, web-based decision aid tools have been developed to help returnees making the decision of disclosing their illness, which already have been found to be helpful [30,31].

The cooperation of rehabilitation physicians/psychotherapists and occupational physicians has been described to be beneficial in improving the RTW of patients [32]. Only little has been known thus far about the possible fear of psychotherapists to have to share information about the mental health of their patients [11]. We addressed this gap by reporting that the psychotherapists are unsure, if information forwarded to the occupational physician could have legal consequences pertaining to the employment contract of the patient. Studies showed that both the occupational physicians and rehabilitation physicians reported to be interested in improving their communication and cooperation while the intensity of communication and cooperation remained low [9,33,34]. Nevertheless, the occupational physician has a unique position due to his/her medical knowledge and the subjection to the medical confidentiality as well as the knowledge of the working conditions of the employees/patients. One concept, which could foster the cooperation of occupational physicians and psychotherapists, relates to so-called psychosomatic consultations in the workplace. These consultations enable easy and fast access to consultations with a psychotherapist which increases the likelihood that CMDs can be detected and treated early [35]. In the Netherlands, collaborative care applied by occupational physicians led to a faster response in terms of reduction in depressive symptoms compared to usual care. Nevertheless, no difference between the two groups has been found related to the duration until RTW, time to remission of depressive symptoms [36]. Therefore, further research on the possible effectiveness of this concept is needed.

The occupational physician has been described as the most important stakeholder in workplace-related prevention (especially tertiary prevention) by different medical and occupational stakeholders (i.e., primary care physicians, psychotherapists and human resource managers) [19]. It is therefore recommended to strengthen the mediating position of the occupational physician in the RTW process to improve the communication between the stakeholders of the RTW process. As it has been shown in previous studies conducted in the Netherlands, the introduction of guidelines addressing the inclusion of occupational physicians in the RTW process is associated with a shortened time to return as well as shortened sickness absence [7,37]. Therefore, the development and actual implementation of guidelines improving the inclusion of occupational physicians into the return to work of employees with CMDs in Germany should be sought and evaluated scientifically.

### 4.2. Strengths and Limitations 

In this study, we carried out qualitative interviews among different stakeholder of the RTW process. Although we completed interviews until thematic saturation was reached, we may not have captured the whole range of potential perspectives. We were not able to recruit patients, supervisors and colleagues working in all three economic sectors (i.e., primary production (first), industrial sector (second), service sector (third)), but we only conducted interviews with employees from the secondary and tertiary sector. We therefore might not have captured the full range of views if one assumes that workers from other sectors may have differing attitudes regarding CMDs. As the first sector comprises the agricultural and forestry sector as well as the extraction of mineral resources (i.e., male-dominated sectors), we might have missed the attitudes of men, who report higher levels of shame due to CMD [38]. Furthermore, we cannot rule out that only interested and particularly committed stakeholders (especially supervisors) took part in this study. Therefore, we cannot exclude a selection bias, which may have restricted the scope of shared views and opinions. Additionally, in terms of social desirability, answers and opinions especially of the supervisors and colleagues may not have reflected reality. Furthermore, due to data protection and ethical reasons, we were not able to recruit direct supervisors or colleagues of the returning patients. We therefore tried to recruit supervisors and colleagues from a wide range of professions to aim a greater range of views. As those participants may have not experienced employees with a CMD we used case vignettes so that the participants could imagine the situation better. Additionally, as the returnees did not nominate many occupational physicians, we conducted interviews with occupational physicians, who were independent from the returnees and their specific situation.

The data have been content-analyzed and revised by scientific researchers of the study team who are experienced in performing qualitative interviews as well as analyzing interviews in the field of social and health sciences as well as the medical profession. Nevertheless, we could not include a second analyst for the final dataset, which could have improved the interpretation. 

## 5. Conclusions

As perspective taking, empathy and the understanding of the other stakeholders’ viewpoint is one of the main factors promoting a successful return, we tried to disclose the mutual expectations of the different stakeholders involved in the RTW process. This knowledge should promote the understanding of the other stakeholders’ aims and to disclose potentially diverging expectations. The main finding pertains to strengthening the mediating role by occupational physicians, who could coordinate the return process due to his/her unique position (medical expertise and medical secrecy as well as workplace-related expertise).

## Figures and Tables

**Table 1:** Study sample of returning employees (*n* = 24).

Characteristics	*n* (%)
Age, mean (standard deviation, SD)	48.8 (9.6)
Female, *n* (%)	10 (41.7)
Family status, *n* (%)	
Married/in a partnership	18 (78.3)
Single/Divorced/Widowed	5 (21.7)
Household with partner, *n* (%)	16 (66.7)
Household with children, *n* (%)	9 (37.5)
Highest professional education, *n* (%)	
Vocational training	13 (56.5)
University degree	7 (30.4)
Other	3 (13.1)
Occupation, *n* (%)	
Primary sector	0 (0.0)
Secondary sector	7 (30.4)
Tertiary sector	16 (69.6)
Permanent contract, *n* (%)	22 (95.7)
Working hours per week, *n* (%)	
≤20	1 (4.3)
20–40	15 (65.3)
≥40	7 (30.4)
Working hours after RTW, *n* (%)	
≤20	0 (0.00)
20–40	16 (76.2)
≥40	5 (23.8)
Desired working hours after RTW, *n* (%)	
Equal amount	16 (69.6)
Less working hours	7 (30.4)
Work requirements, *n* (%)	
Physical	2 (8.7)
Mental	15 (65.2)
Both equally	6 (26.1)
Work ability, mean (SD) ^1^	5.1 (2.8)
Location of interview, *n* (%)	
Inpatient rehabilitation clinic	12 (50)
Daycare rehabilitation clinic	8 (33.3)
Company	4 (16.7)
Medical diagnosis depression/burnout, *n* (%)	23 (95.8)
Duration of incapacity to work, *n* (%)	
7–11 weeks	2 (9.1)
3–6 months	5 (22.7)
>6 months	15 (68.2)
Professional assistance (multiple responses allowed)	
General practitioner	15
Psychological psychotherapist	16
Psychiatrist	14
Inpatient psychiatry	7
Psychosomatic rehabilitation	17
Mental health, mean (SD) ^2^	5.4 (1.9)
Shame due to own mental illness, mean (SD) ^3^	3.0 (2.0)
I do not want contact to colleagues with mental illness, mean (SD) ^4^	1.9 (1.7)
Colleagues and superiors with mental health problems are supported and treated fairly, mean (SD) ^4^	5.0 (2.5)
I would disclose my mental illness to my colleagues, mean (SD) ^5^	4.1 (2.8)

^1^ scale ranging from 0 = completely unable to work to 10 = currently the best work ability; ^2^ scale ranging from 1 = completely mentally healthy to 9 = mentally seriously ill; ^3^ scale ranging from 1 = not at all to 9 = very strongly; ^4^ scale ranging from 1 = not at all to 9 = totally agree; ^5^ scale ranging from 1 = certainly not to 9 = certainly.

**Table 2:** Study sample of health care professionals (*n* = 26).

Characteristics	*n*(%)
Age, mean (SD)	51.6 (10.4)
Female, *n* (%)	14 (53.8)
Specialization (multiple responses allowed)	
General practitioner	8
Occupational physicians	13
Psychological psychotherapist	6
Medical psychotherapist	3
Psychiatrist	3
Psychological psychotherapist in training	3
Social worker (rehabilitation clinic)	1
Work experience (years), mean (SD)	15.6 (9.0)

**Table 3:** Study sample of supervisors and colleagues (*n* = 18).

Characteristics	*n* (%)
Age, mean (SD)	50.3 (10.1)
Female, *n* (%)	9 (50.0)
Highest professional education, *n* (%)	
Vocational training	4 (25.0)
University degree	10 (62.5)
Other	2 (12.5)
Occupation, *n* (%)	
Primary sector	0 (0.0)
Secondary sector	0 (0.0)
Tertiary sector	18 (100)
Leading position, *n* (%)	8 (50.0)
Working hours per week, *n* (%)	
≤20	0 (0.0)
20–40	11 (68.8)
≥40	5 (31.2)
Main type of work demands, *n* (%)	
Physical	0 (0.0)
Mental	13 (81.2)
Both equally	3 (18.8)
Work ability, mean (SD) ^1^	9.6 (1.3)
Incapacity to work due to mental illness (past 12 months), *n* (%)	0 (0.0)
Mental health, mean (SD) ^2^	2.1 (0.8)
I do not want contact to colleagues with mental illness, mean (SD) ^3^	2.3 (1.7)
Colleagues and superiors with mental health problems are supported and treated fairly, mean (SD) ^3^	6.3 (1.8)
I would disclose my mental illness to my colleagues, mean (SD) ^4^	5.6 (2.1)

^1^ scale ranging from 0 = completely unable to work to 10 = currently the best work ability; ^2^ scale ranging from 1 = completely mentally healthy to 9 = mentally seriously ill; ^3^ scale ranging from 1 = not at all to 9 = totally agree; ^4^ scale ranging from 1 = certainly not to 9 = certainly.

## Supplementary Materials

The following are available online at https://www.mdpi.com/1660-4601/17/18/6635/s1, Table S1: Quotes.

## References

[1] Ahola K., Virtanen M., Honkonen T., Isometsä E.T., Aromaa A., Lönnqvist J. (2011). Common mental disorders and subsequent work disability: A population-based Health 2000 Study. J. Affect. Disord..

[2] Ferrari A.J., Charlson F.J., Norman R.E., Flaxman A.D., Patten S.B., Vos T., Whiteford H.A. (2013). The Epidemiological Modelling of Major Depressive Disorder: Application for the Global Burden of Disease Study 2010. PLoS ONE.

[3] Wege N., Angerer P. (2013). Psychische Erkrankungen—Auswirkungen auf die Arbeitsfähigkeit und Versorgung psychisch erkrankter Erwerbstätiger. Psychiatrie.

[4] OECD/EU (2018). Health at a Glance: Europe 2018: State of Health in the EU Cycle.

[5] De Vries H., Fishta A., Weikert B., Sanchez A.R., Wegewitz U. (2018). Determinants of Sickness Absence and Return to Work Among Employees with Common Mental Disorders: A Scoping Review. J. Occup. Rehabilitation.

[6] Dewa C.S., Trojanowski L., Joosen M.C.W., Bonato S. (2016). Employer Best Practice Guidelines for the Return to Work of Workers on Mental Disorder–Related Disability Leave. Can. J. Psychiatry.

[7] Nieuwenhuijsen K., Faber B., Verbeek J.H., Neumeyer-Gromen A., Hees H.L., Verhoeven A.C., Van Der Feltz-Cornelis C.M., Bültmann U. (2014). Interventions to improve return to work in depressed people. Cochrane Database Syst. Rev..

[8] Franche R.-L., Baril R., Shaw W., Nicholas M., Loisel P. (2005). Workplace-Based Return-to-Work Interventions: Optimizing the Role of Stakeholders in Implementation and Research. J. Occup. Rehabilitation.

[9] Stratil J.M., Rieger M.A., Mahlknecht U. (2017). Cooperation between general practitioners, occupational health physicians, and rehabilitation physicians in Germany: What are problems and barriers to cooperation? A qualitative study. Int. Arch. Occup. Environ. Heal..

[10] Moßhammer D., Natanzon I., Manske I., Grutschkowski P., Rieger M.A. (2013). Cooperation between general practitioners and occupational health physicians in Germany: How can it be optimised? A qualitative study. Int. Arch. Occup. Environ. Heal..

[11] Rothermund E., Michaelis M., Jarczok M.N., Balint E.M., Lange R., Zipfel S., Gündel H., Rieger M.A., Junne F. (2018). Prevention of Common Mental Disorders in Employees. Perspectives on Collaboration from Three Health Care Professions. Int. J. Environ. Res. Public Heal..

[12] Stegmann R., Schröder U.B. (2016). Psychische Erkrankungen in der Arbeitswelt: Wiedereingliederung nach einer psychischen Krise. Ergebnisse einer qualitativen Studie. ASU.

[13] Longmire N.H., A Harrison D. (2018). Seeing their side versus feeling their pain: Differential consequences of perspective-taking and empathy at work. J. Appl. Psychol..

[14] Galinsky A.D., Maddux W.W., Gilin D., White J.B. (2008). Why it pays to get inside the head of your opponent: The differential effects of perspective taking and empathy in negotiations. Psychol. Sci..

[15] Sturges J.E., Hanrahan K.J. (2004). Comparing Telephone and Face-to-Face Qualitative Interviewing: A Research Note. Qual. Res..

[16] Schonfeld I.S., Mazzola J.J., Sinclair R.R., Wang M., Tetrick L.E. (2012). Strenghts and limitations of qualitative approachs to research in occupational health psychology. Research Methods in Occupational Health Psychology.

[17] Scharf J., Nguyen X.Q., Vu-Eickmann P., Krichbaum M., Loerbroks A. (2018). Perceived Usefulness of Continuous Glucose Monitoring Devices at the Workplace: Secondary Analysis of Data From a Qualitative Study. J. Diabetes Sci. Technol..

[18] Völter-Mahlknecht S., Rieger M.A. (2014). Patient care at the interface between rehabilitation and occupational health physicians—a systematic literature review focusing health care organization. Dtsch. Med. Wochenschr..

[19] Michaelis M., Balint E.M., Junne F., Zipfel S., Gündel H., Lange R., Rieger M.A., Rothermund E. (2019). Who Should Play a Key Role in Preventing Common Mental Disorders that Affect Employees in the Workplace? Results of a Survey with Occupational Health Physicians, Primary Care Physicians, Psychotherapists, and Human Resource Managers. Int. J. Environ. Res. Public Heal..

[20] Dietrich S., Mergl R., Rummel-Kluge C., Stengler K. (2012). Psychische Gesundheit in der Arbeitswelt aus der Sicht von Betriebs- und Werksärzten. Psychiat. Prax..

[21] Lemieux P., Durand M.-J., Hong Q.N. (2011). Supervisors’ Perception of the Factors Influencing the Return to Work of Workers with Common Mental Disorders. J. Occup. Rehabilitation.

[22] Negrini A., Corbière M., LeComte T., Coutu M.-F., Nieuwenhuijsen K., St-Arnaud L., Durand M.-J., Gragnano A., Berbiche D. (2017). How Can Supervisors Contribute to the Return to Work of Employees Who have Experienced Depression?. J. Occup. Rehabilitation.

[23] Andersen M.F., Nielsen K.M., Brinkmann S. (2012). Meta-synthesis of qualitative research on return to workamong employees with common mental disorders Scand. J. Work Environ. Health.

[24] Shaw W.S., Robertson M.M., Pransky G., McLellan R.K. (2003). Employee perspectives on the role of supervisors to prevent workplace disability after injuries. J. Occup. Rehabilitation.

[25] Bethge M. (2016). Effects of graded return-to-work: A propensity-score-matched analysis. Scand. J. Work. Environ. Heal..

[26] Joyce T., Higgins I., Magin P., Goode S.M., Pond D., Stone T., Elsom S., O’Neill K. (2012). The Experiences of Nurses With Mental Health Problems: Colleagues’ Perspectives. Arch. Psychiatr. Nurs..

[27] Marino C.K., Child B., Krasinski V.C. (2016). Sharing Experience Learned Firsthand (SELF): Self-disclosure of lived experience in mental health services and supports. Psychiatr. Rehabilitation J..

[28] Munir F., Leka S., Griffiths A. (2005). Dealing with self-management of chronic illness at work: Predictors for self-disclosure. Soc. Sci. Med..

[29] Brouwers E.P.M., Joosen M.C.W., Van Zelst C., Van Weeghel J. (2019). To Disclose or Not to Disclose: A Multi-stakeholder Focus Group Study on Mental Health Issues in the Work Environment. J. Occup. Rehabilitation.

[30] Stratton E., Choi I., Calvo R., Hickie I., Henderson C., Harvey S., Glozier N. (2019). Web-based decision aid tool for disclosure of a mental health condition in the workplace: A randomised controlled trial. Occup. Environ. Med..

[31] Chakraverty V., Bauer J.F., Jakob L., Niehaus M. (2018). Arbeitnehmer mit chronischen Erkrankungen im Entscheidungsdilemma—Individuelle Unterstützung durch eine digitale Reflexionshilfe zur Offenbarung gesundheitlicher Beeinträchtigungen im Arbeitsleben. Psychother. Psychosom. Med. Psychol..

[32] Schwarze M., Spallek M., Korallus C., Manecke I.-A., Teumer F., Wrbitzky R., Gutenbrunner C., Rebe T. (2012). Advantages of the JobReha discharge letter: An instrument for improving the communication interface in occupational rehabilitation. Int. Arch. Occup. Environ. Heal..

[33] Seidel H., Neuner R., Schochat T. (2003). Occupational health physician and medical rehabilitation—A survey among occupational health physicians in Baden-Wuerttemberg. Arbeitsmedizin Sozialmedizin Umweltmed.

[34] Vroeijenstijn-Nguyen X., Brenner R. (2007). Contact between occupational health physicians and rehabilitation physicians—todays reality for a better future?. Tijdschr Bedr Verzek.

[35] Preiser C., Rothermund E., Wittich A., Gündel H., Rieger M.A. (2015). Psychosomatic consultation in the workplace: Opportunities and limitations of the services offered—results of a qualitative study. Int. Arch. Occup. Environ. Heal..

[36] Vlasveld M.C., Van Der Feltz-Cornelis C.M., Adèr H.J., Anema J.R., Hoedeman R., Van Mechelen W., Beekman A.T.F. (2012). Collaborative care for sick-listed workers with major depressive disorder: A randomised controlled trial from the Netherlands Depression Initiative aimed at return to work and depressive symptoms. Occup. Environ. Med..

[37] Van Der Klink J.J.L., Blonk R.W.B., Schene A.H., Van Dijk F.J.H. (2003). Reducing long term sickness absence by an activating intervention in adjustment disorders: A cluster randomised controlled design. Occup. Environ. Med..

[38] Hahm S., Speerforck S., Fleischer T., Grabe H.J., Beutel M., Schomerus G. (2020). The Effects of Gender, Education, and Income on Anticipated Shame Regarding Mental Illness—Results of a German Population Study. Psychiat. Prax..

