# Supplementary material for: Return to Work after Common Mental Disorders: A Qualitative Study Exploring the Expectations of the Involved Stakeholders
Source: Int J Environ Res Public Health. 2020 Sep 11;17(18):6635. doi: 10.3390/ijerph17186635 (PMC7558125; doi:10.3390/ijerph17186635)
Supplement: Supplementary file 1 [file ijerph-17-06635-s001.pdf]

Table S1. Quotes.

1	I know he exists. I think I have seen him twice. But I don't think he plays a role in this context. […] I think the occupational physician is only, when it comes to finding out if the physician's diagnosis is correct, or something like that, that he checks this again. P15<br><br>If there was simply an information, I find this totally OK. This could be a brief, short statement like „We would like to inform you about this opportunity, in case you would like to use it". And then the contact information. P23
2	We do have an occupational PHYSICIAN SOMEWHERE but he's not always there. One does have to make a request. P3
3	Somehow nothing happens and so on. And then she let me know „Hey, sorry, Mr X, the things that were discussed during the conversation concerning BEM, no-one cares about them at all. If the immediate supervisor says no, then it's no, period." That's my experience with a conversation about BEM. P22
4	WHAT I would find quite HELPFUL, I think, would be just be having a kind of toolbox or checklist that provides a selection of options depending on the disease: What type of measures could help? Or what, type of measures have helped others? […] And THAT'S of course where some type of support would actually be good, if some kind of best practices, as we say in neo-German where one simply says: What has already helped others? What helped in other SITUATIONS? That one collects them and looks at them together: What seems best to me? Or what fits the problems one has best? I think that would really be helpful P15
5	Perhaps one should write it all down. So that conversations on BEM should begin BEFORE reintegration instead of afterwards. AND, as I said, what was written down, one should stick to it. P4
6	If he hesitates, then I often tell him to sleep on it for two nights and to think about it then. Because to start with BEM without being sufficiently motivated, I think that's not good. It's frustrating for both sides. On the one hand for the employee because he won't move on, and also for the employer because nothing fits. OP1<br><br>I: In your opinion who is the most responsible person or contact person? B: Well, basically the employee himself, right? Because the employee himself is the actor in the conversation about BEM, the one who can decide a bit for himself. OP4
7	Actually, I don't know in advance that patients are mentally ill. They may let me know me during the appointment that's our first contact. OP6
8	I will give you an example: There are many hidden objectives, from the employer as well as from the employee himself and often reintegration fails because it's not about the disease but about hidden objectives and questions about power and influence, who decides, who determines what will happen and what will not happen? And in such a specific case, especially if the occupational physician has insufficient knowledge of the history, if neither side is open, neither the employer nor the employee, then it will be extremely difficult for the occupational physician to initiate something useful. OP9
9	Expectation of the person who returns is to be flexible. That they do not expect that all their wishes will be fulfilled. And that they understand that some jobs need to be done even if it is harder for them than for others. OP10
10	Well patients are treated with kid gloves a lot. And they are very sensitive when it comes to how you treat them. It strikes me more about the mentally ill. "My boss wasn't very happy when I returned". And then they arrive frustrated and complain. That's also something where you can help getting a new perspective. "Well, the time without you wasn't easy for him as well." Most employees within BEM who return because of a mental illness were absent for a very, very long

	time, somewhere around half a year. OP6
11	Basically, I wish there were independent levels THAT for example could act preventively, that for example partly cooperate with the company, to which the employee can turn without feeling or WORRYING: "I will lose my job if I share information openly." (S16)
12	Expectations of the supervisors are simply that they are really interested in the process of reintegration. And not simply want to get rid of the sick employee. OP9
13	Being interested and thus collecting information. Well I don't know, if for example education on mental illnesses for executive personnel or something like that, I am not sure how many people would register. Well, from my point of view there should be something like a leadership license anyway. Everyone having a leadership role should have a certain number of tools and skills so that this will be successful. Well, normally this isn't the case. OP9
14	There again, if possible, to deal with mental illnesses without any prejudices. This is a big, big concern for me. (OP5); If the workplace doesn't fit, then to be flexible and act accordingly. There are some supervisors who are a bit narrow-minded in this respect. OP1
15	Of course in a team, the team he belongs to, if he returns there, sometimes or in most cases we create new jobs, then I of course expect some understanding that the person concerned is not as quick, as fast. First, he needs to become acquainted with the new job, and then he's just at the beginning and can't go full speed. Well that's what I expect from the persons concerned. But it's my impression that the supervisors support this and provide protection accordingly. OP1
16	Expectations that agreements, as far as they were made, will be observed and that people cooperate in realizing reintegration with a certain goodwill. OP9
17	Because finding the balance between protection and excessive demands, to find that, you can only do it with empathy. And I think we have not only managers with many years, that especially skills like putting oneself in someone's position, being empathetic sometimes without abandoning expectations, that this is not very pronounced. OP6
18	I think it is important that a supervisor, I myself was one for a long time, IF something like that happens, that on the first day one really spends three or four hours talking to the employee. It doesn't have to be three or four hours, it can also be two. That one really takes time and says: "Once you return, let's start by talking. Just as an update." P14
19	What I do NOT want is that they put pressure on me by telling me: "Mr. X, it must be done by then. And if this doesn't work, then that's bad luck for you, we will fire you." P1<br><br>I think it's important that it's not immediately: "Now your back, let's go full speed." That the company starts by showing sympathy, that in the beginning they don't put too much work on my table, but say instead: "Now do THIS and that's enough for today. We'll go on tomorrow." That you get re-introduced slowly. P7
20	Well, it was excellent that there were no problems with reintegration. This means, today I can manage four hours well, next I will also be able to do six hours. And once my self-confidence is back and I SEE successes, I have succeeded, then I can go back to fulltime. P14
21	And then someone asked me: "WHAT would help you most." Because they certainly don't know. Because how could they know? And THAT'S of course where some type of support would actually be good, if some kind of best practices, as we say in neo-German, where one simply says: What has already helped others? What helped in other SITUATIONS? That one collects them and looks at them together: What seems best to me? Or what fits the problems one has best? I think that would really be helpful. P15
22	So that one, let me say it like this, plays at cards with honesty. Instead of generating fanciful ideas that people rely on, then nothing happens. P4

23	AND of course openness, if he then sometimes says "I can't do it right now, or right now I'm feeling overwhelmed, or I need a half-hour break now", then one needs to address it openly. Then I can deal with it BETTER than when nothing is said and the work is badly done or something like that, right? I think being open is very important. S3<br><br>But I do think that someone who COOPERATES has a lot of leeway. […] I do believe that this will be supported by the team. S4<br><br>As I said, it is NOT necessary to give access to the medical records, but I think open communication to say this is what I can still do, I will be able to do this AGAIN but I can't do it at the moment, I think that's best for all parties involved. S14
24	And thirdly, if I know what I expect or can expect from a colleague, then I can make plans accordingly, right? And then I also know how far I can go and what I can demand and what and where the limits are. If I know that and can estimate it, then that's perfectly fine. (S10); Sure, then there's less understanding, if you don't know that, as when someone says: "Hey, I had this and that problem." That's like if someone broke a leg, then you don't expect him to run a marathon. If you know about it, that's OK. But if you don't know it, then you expect more of him, than if you do. And that's not good for him. But one doesn't know that. S12
25	Well, at least I ask if he will still be willing to receive professional support on a private basis? I always find this very, very important. That's what I expect. Not that you reach the point of saying: "I am in good health, I can do everything on my own." Usually that happens rarely. That he continues to, receive assistance. No, not assistance, that's the wrong word, professional support. S9
26	The question is also how LONG should the understanding take? Well, at some point in time the employee, that's what I assume, will be more or less fully involved. And then the QUESTION is he healthy again then? Or do I STILL have to be considerate of something? C3<br><br>And if THIS then, all the tasks will be upon me, then this would of course be an additional burden for me. And then I would probably seek the conversation and say: "If you can't do this, then I can't take over all of this. Perhaps our boss needs to do it." Or one has to redistribute it. C3<br><br>If one SUPPORT is missing, the other can't fully replace it. Fully, or for the most part. And I think that's when the supervisors need to act. Well, BECAUSE in the worst case the situation evolves that the other one also becomes overloaded and is also unavailable. And then it is, well this would be the worst case. C3
27	In my opinion it is important to share OPENLY WHY this is how it works right now. And that the team leaders or supervisors acknowledge that as a colleague one has to work more over a certain period of time and that they appreciate it. For me it is very important. C14
28	That the employees, the colleagues understand it. Sure, if someone is not available and it's impossible to hire a new person, then the others will take over the tasks. Of course, that's always difficult, to build some understanding. S11<br><br>A supervisor must put VERY, very much effort into persuasion, to say to his own bosses but also within the team of the organizational unit: "Now you don't' have to point your finger at someone, let's be happy that he's even there and that he can work for eight HOURS. And if that's how we want it to continue, then we'll leave it at that." I imagine this would be difficult. S7
29	One doesn't even have to say that one is ILL. Or rather the others, they're not supposed to know. But in order to get support of the team and ask for understanding, I would say it would make sense. And for MYSELF as a team colleague it would be helpful to be able to assist and support the colleague. C3
30	The question is also how LONG should the understanding take? Well, at some point in time the employee, that's what I assume, will be more or less fully involved. And then the QUESTION is he healthy again then? Or do I STILL have to be considerate of something? And there I would say, then I think I would probably like to have him as a fulltime employee of course, or have him ON BOARD

	as a fulltime employee. Then I would probably be less considerate. C3<br><br>But if my impression is that there is no end. Then at some point I reach my limit. Well, I can carry an extra load for a certain amount of time without problems. I am very, very resilient. But if my impression is that my resilience reaches its limit and I don't think that this will END at some point, and I don't have the impression that my supervisor will intervene at some point, THEN I have to put on the brakes and think of myself. Because I can't forget myself only because I want to help others. C14
31	That one does have the impression the person concerned MAKES AN EFFORT and doesn't use the illness as an excuse. C14
32	With sympathy, that one says: "It's great that you're back. Now let's see how we can manage that." That my wishes will be taken into account, too, and a solution will be found everyone can live with. That people react stupidly: "What, you need a break" or something similar, but that they sympathize and say "Yes, of course, that's no problem if it makes you feel better." Something like that. P7
33	I hope that they just deal with the issue openly. On the other hand I hope that they don't react by saying: OK, we need to go easy on him now. Or we mustn't burden him. I would NOT want that. Because I also find it TERRIBLE when there are NOT ENOUGH challenges and there's some support for all tasks. I find that difficult. Well, what I REALLY want is normality. P21<br><br>They SHOULD just treat me as a normal team member, as they used to do. P14<br><br>That one acts a bit carefully and approaches him carefully but doesn't get on his NERVES; just ask him: "Can I help you" Or "If you have a question, there are no stupid questions, just ask me." Offer something like that. P16<br><br>Well, that's why I expect a bit, that at least in the beginning there will be a period of grace. P11
34	Well, a bit of sympathy for my going a bit slower. Then I will be back to normal, I presume. P17
35	Well, my therapist, I have to say, she said what I did in therapy would not really help me. She stopped it then. And I wish there would have been more patience. Because now I need to find someone new. P24
36	I have the support. And I think the psychiatrist has already sent a letter […] But he said he sent a letter that this workplace is no longer suitable for me. And I think that has MORE influence than when the psychiatrist writes it, or the psychologist. Because if they see he was in a psychosomatic hospital then that's perhaps an extra step. P4<br><br>Perhaps, if the psychiatrist or the psychologist writes some kind of letter that states it accordingly, that this will be used as a basis. Or makes a recommendation, how it could be better, that one could take along. P7
37	EXPECTATIONS, that's always the case, to take this STEP and to return to work. Even if it is only as part of the reintegration. Patients HAVE a lot of anxiety and doubts and: "Will I really be able to make it?" EXPECTATIONS in the sense to motivate them to take this step. To nudge them a bit. And at some point, you have to return to reality. And to ENCOURAGE them, that's how I would formulate it. T(P5)
38	Well, I think what I would of course expect, or HOPE FOR, is that we talk about it openly or in a transparent manner. […] I think this implies that I can provide some support for a patient during reintegration at the workplace, that he is open about it and shares his FEARS, worries with me. Because it's only then that one can look at how to deal with it accordingly. And perhaps TAKE AWAY some fears, and say again: "Look, they mean you no HARM, people here want to support you. Sure, they act in the company's interest, but of course also in yours." And yes, I think this is a very great expectation. T(P20)

39	I DO expect from patients, that they understand how they THEMSELVES contribute to the evolution of the problem. Patients who say. “It’s ONLY the others!”, and that happens quickly, sometimes it’s understandable. T(P14)<br><br>There are situations in which a person doesn’t really want to change and is rather RIGID in his course of action and impulses, from inside, to change somehow. Then this will ALSO be difficult. But most of the problems exist because the system around these people can’t be fundamentally changed. T(P9)
40	I expect that he does try to do his best. To apply the strategies that we developed. And puts things we developed in therapy INTO PRACTICE. T(P22)
41	THAT would be such an improvement, if at the same time there wasn’t only the GP’s application, the application for rehabilitation, but also a short letter from the occupational physician. T(P8+9)
42	Well, the advantage is, if an occupational physician gets involved, that he knows the situation onsite, knows the WORKplace so to speak. Whereas as a GP one only knows about the workplace from the patient’s point of VIEW. That’s how he sees it and describes it, and perhaps that doesn’t always correspond to reality 100%. In that respect someone who knows the actual situation very WELL can perhaps make a better judgement. T(P23)<br><br>I could, but so far it hasn’t been necessary because patients have always directly provided me with the information. T(P13)
43	If the patient agrees to it. It probably depends on confidentiality. And that’s where it begins, if a relationship at a workplace is very conflicted, then it’s possible that they don’t WANT to. It is sometimes a question: Could it jeopardize an employment relationship? These would be LEGAL aspects and it would be interesting if this is something that could be done at all. I wonder, I don’t know. T(P6)
44	I wouldn’t do that, the local GP would do it. In rare cases she will contact the occupational physician. BECAUSE in such cases, I must ALSO point that out critically, occupational physicians resent to make a phone call to social workers in a hospital. Because they always only want to call physicians. T(P17)<br><br>Well, what would be necessary is that we both get on the phone and TALK to one another. Well that’s not a big deal. But the problem OFTEN is lack of time, because it’s not only difficult about such things, but it’s generally the case that many therapists haven’t quite understood yet that it makes sense to exchange information. Well in my opinion it’s not a big EFFORT, but often there is not enough time to deal with it in between. T(P22)
45	Sometimes it was my impression, it depends on whether they work more in psychotherapy or in behavior therapy that they are far away from the professional reality of the diseased. Sometimes in exceptional cases I have seen individuals who have been in psychotherapy, especially support based on depth psychology, and there the job situation and social security and things like that weren’t taken into account at all. OP9
46	Exaggerated expectations, wrong expectations. If one states: “The company is big, they’ll find something for you.” That we of course still have a problem to find something, that’s not significantly better in a large company than in a small company. Nobody cheers if he’s expected to take over someone new who is ill. And that’s perhaps even more difficult for a mental illness then for an internal disorder. OP6
47	Perhaps also that he has a clear view for the realities in the company. For example, something that is difficult or problematic for us is when psychotherapists think that at a certain diagnosis certain things aren’t possible. For example, someone who has suffered from depression at some point should no longer work in alternating shifts. […] There’s this one workplace, and it includes alternating shifts, or he would indeed have to, if he is no longer able to work in alternating shifts, to change the workplace, in a completely new department and to start from scratch. And that’s an

	issue, that that's hard. OP10

Abbreviations: OP: occupational physician, P: patient (P1 is the first patient interviewed), T(P1): therapists (nominated therapist of patient one), S: supervisor and C: colleagues
